# Computed tomography and magnetic resonance imaging observations of rhabdomyosarcoma in the head and neck

**DOI:** 10.3892/ol.2014.2094

**Published:** 2014-04-25

**Authors:** JINGQI ZHU, JIANHUA ZHANG, GUANGYU TANG, SHIYOU HU, GUOXING ZHOU, YONGKANG LIU, LINGLING DAI, ZHONGQIU WANG

**Affiliations:** 1 Department of Radiology, Shanghai Tenth People’s Hospital, Tongji University School of Medicine, Shanghai 200072, P.R. China; 2 Department of Radiology, East Hospital, Tongji University School of Medicine, Shanghai 200120, P.R. China; 3 Department of Oncology, Affiliated Hospital of Nanjing University of Traditional Chinese Medicine, Nanjing, Jiangsu 210029, P.R. China; 4 Department of Radiology, Affiliated Hospital of Nanjing University of Traditional Chinese Medicine, Nanjing, Jiangsu 210029, P.R. China

**Keywords:** rhabdomyosarcoma, magnetic resonance imaging, head and neck, computed tomography

## Abstract

Head and neck (HN) rhabdomyosarcoma (RMS) is an aggressive malignancy, which is rarely encountered and is commonly misdiagnosed as another type of tumor. The aim of the present study was to investigate the computed tomography (CT) and magnetic resonance imaging (MRI) features of HNRMS and analyze the correlations between the imaging observations and the pathological subtypes. A total of 10 HNRMS patients (three males and seven females; median age, 16 years) were reviewed retrospectively by only CT (n=1), only MRI (n=2), as well as CT and MRI (n=7). In addition, the clinical data, imaging observations and pathological results were recorded and analyzed. The origins of the 10 HNRMSs (eight embryonal and two alveolar subtypes) included the ethmoid sinus (n=4), maxillary sinus (n=1), orbit (n=3), nasopharynx (n=1) and frontotemporal subcutaneous area (n=1). On the CT and MRI images, the soft-tissue masses exhibited ill-defined borders (n=9), bony destruction (n=10), multi-cavity growth (n=7) and cervical lymph node metastasis (n=2), whereas calcification and hemorrhaging were not identified. On CT, eight of the HNRMSs appeared slightly hypodense (2/8) or isodense (6/8) with homogeneous enhancement (4/4). On T1-weighted images (WI), nine tumors exhibited isointensity (9/9) and on T2WI, six tumors demonstrated homogeneous hyperintensity with homogeneous enhancement on contrast-enhanced (CE)-T1WI. In addition, three embryonal RMSs, which originated from the ethmoid sinus, exhibited heterogeneous hyperintensity on T2WI and nodule-shaped enhancement patterns on CE-T1WI. The results of the present study indicated that MRI may accurately demonstrate the location and extent of HNRMS and that the imaging features of HNRMS may be similar to those of other tumors. However, a tumor exhibiting heterogeneous hyperintensity on T2WI and a nodule-shaped enhancement pattern on CE-T1WI in the ethmoid sinus may present specific MRI features, which clearly indicates the botryoid subtype of embryonal RMS.

## Introduction

Rhabdomyosarcoma (RMS) is a rare and aggressive malignancy possibly originating from primitive mesenchymal cells that arise anywhere in the body, including sites where striate muscle is not found (1). The annual incidence of RMS in children is reported to be 4.3 cases per million (2). RMS is the most common type of soft-tissue sarcoma in young children, representing 5% of all childhood malignancies (3). By contrast, RMS occurs less frequently in adults (4).

Almost half of RMS occur in the head and neck (5–8) and three different primary sites of head and neck RMS (HNRMS) have been recognized in the following locations: parameningeal (PM), non-PM (NPM) and orbital (ORB) (9). In addition, surviving hereditary retinoblastoma patients have an increased risk of craniofacial second primary tumor (SPT), such as RMS, particularly following treatment with external beam radiotherapy. RMS is one of the most common types of craniofacial SPT in irradiated hereditary retinoblastoma patients, which develops in specific locations (such as the ethmoid sinus and temporal fossa) (10). HNRMS is commonly confused with other types of rapidly progressive malignant tumors of the head and neck, including lymphoma, nasopharyngeal carcinoma (NPC), primitive neuroectodermal tumors, Langerhans cell histiocytosis, olfactory neuroblastoma (ONB), osteosarcoma and metastasis (1,10–13).

The aim of the present study was to investigate the computed tomography (CT) and magnetic resonance imaging (MRI) features of HNRMS and analyze the correlation between the imaging observations and the pathological subtypes.

## Patients and methods

### Subjects

Patients who underwent treatment for RMS at the East Hospital affiliated to Tongji University School of Medicine (Shanghai, China) between 2007 and 2013 were identified from the pathology and health record databases in agreement with the recommendations of the East Hospital ethics committee. The following inclusion criteria were used: i) Availability of adequate CT or MRI information; and ii) histopathological confirmation of RMS. A total of 10 HNRMS patients (three males and seven females; median age, 16 years), who were histologically diagnosed by biopsy (n=8) or surgery (n=2), were included in this retrospective study. The patients had no medical history of hereditary retinoblastoma or treatment with radiotherapy. In addition, their age, gender, symptoms and pathological subtype were recorded.

### CT and MRI technique

In patients with HNRMS, CT is predominantly performed to assess for the absence or presence of bony destruction, calcification and lung metastases. Eight patients underwent CT using a 64-slice spiral CT system (Philips Brilliance; Philips Medical Systems, Best, The Netherlands). The CT scanner parameters were as follows: 250 mAs; 120 kVp; rotation time, 0.75 sec; pitch, 1.204; 25-cm field of view; matrix size, 512×512; slice thickness, 1.5 mm; and detector configuration, 64×0.625 mm. In addition, dual-phase dynamic enhanced scanning (30 and 65 sec) was performed in four patients to obtain images of the arterial and venous stages following the intravenous administration of the contrast agent (Omnipaque 300; 300 mgI/ml; dose, 1.5 ml/kg body weight; injection rate, 2.5 ml/sec) purchased from Nycomed Amersham (Princeton, NJ, USA).

In total, nine patients underwent MRI using a 3.0-Tesla system (Philips Achieva; Philips Medical Systems) and a combined head and neck coil. The parameters of the MRI scanner were as follows: 23-cm field of view; matrix size, 256×192; and slice thickness, 3 mm. T1-weighted spin-echo (SE) images were obtained in the axial plane [repetition time (TR)/echo time (TE), 279/2.3 msec of two excitations]. In addition, T2-weighted fast SE images (TR/TE, 3,118/80 msec of one excitation) and T2-weighted short time inversion recovery in the axial and coronal planes were obtained prior to injection of the contrast material. Following the intravenous administration of gadopentetate dimeglumine (Gd-DTPA: Magnevist^®^; Bayer Schering Pharma AG, Berlin, Germany; dose, 0.1 mmol/kg body weight; injection rate, 1.5 ml/sec), fat-saturated T1-weighted SE images were obtained in the axial, coronal and sagittal planes with the same parameters that were used prior to the Gd-DTPA injection. In seven out of the 10 HNRMS cases, CT and MRI were available.

### Image interpretation

On CT examination, the attenuation of each tumor was recorded as hypo-, iso- or hyperdense as compared with the adjacent muscle. On MRI, the signal intensity of each tumor was recorded as hypo-, iso- or hyperintense as compared with the adjacent muscle.

Two radiologists (specialists in head and neck imaging), who were blinded to the diagnosis of HNRMS, independently evaluated the CT and MRI images and were in agreement. The tumor characteristics, including site, size, margin, local extent, calcification, hemorrhaging, bony destruction and site of metastasis, were recorded. In addition, the attenuation and intensity, as well as the contrast enhancement pattern of the HNRMS were evaluated.

The CT and MRI features together with the clinical data of the 10 HNRMS patients were analyzed using the pathological subtypes. All patients provided written informed consent for participation in the study and for the review of their medical records.

## Results

### Clinical features

The 10 patients (three males and seven females) ranged in age between five and 77 years (median age, 16 years) and 70% of the patients were aged <20 years. The clinical symptoms were not specific, however, they were associated with the tumor site, which included nasal obstruction (n=5), purulent nasal discharge (n=3), proptosis (n=3), visual disturbance (n=2), epistaxis (n=1), hyposmia (n=1) and subcutaneous mass (n=1).

### CT and MRI observations

**Figure 1:**
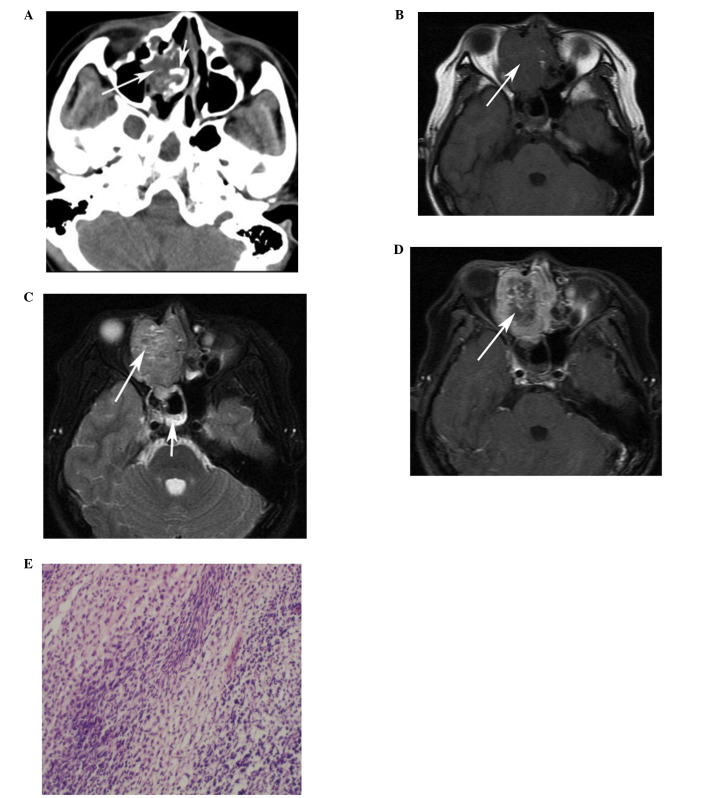
Case 1, a 14-year-old female with embryonal rhabdomyosarcoma in the ethmoid sinus. (A) Axial computed tomography demonstrates a slightly hypodense mass (long arrow) originating from the right ethmoid sinus and extending outside the sinus. Bony destruction with sclerosis (short arrow) is well demonstrated. (B) Axial T1-weighted images (WI) shows the mass (arrow) with homogeneous isointensity and (C) axial T2WI differentiates the heterogeneously hyperintense mass (long arrow) from the significantly hyperintense inflammatory secretion (short arrow) in the ephenoid sinus. (D) Axial contrast enhanced-T1WI shows nodule-shaped enhancement of the mass (arrow). (E) Photomicrograph of the histological specimen demonstrates the spindle-shaped and oval cells with eosinophilic cytoplasm and eccentric nuclei (hematoxylin and eosin stain; magnification, ×100).

**Figure 2:**
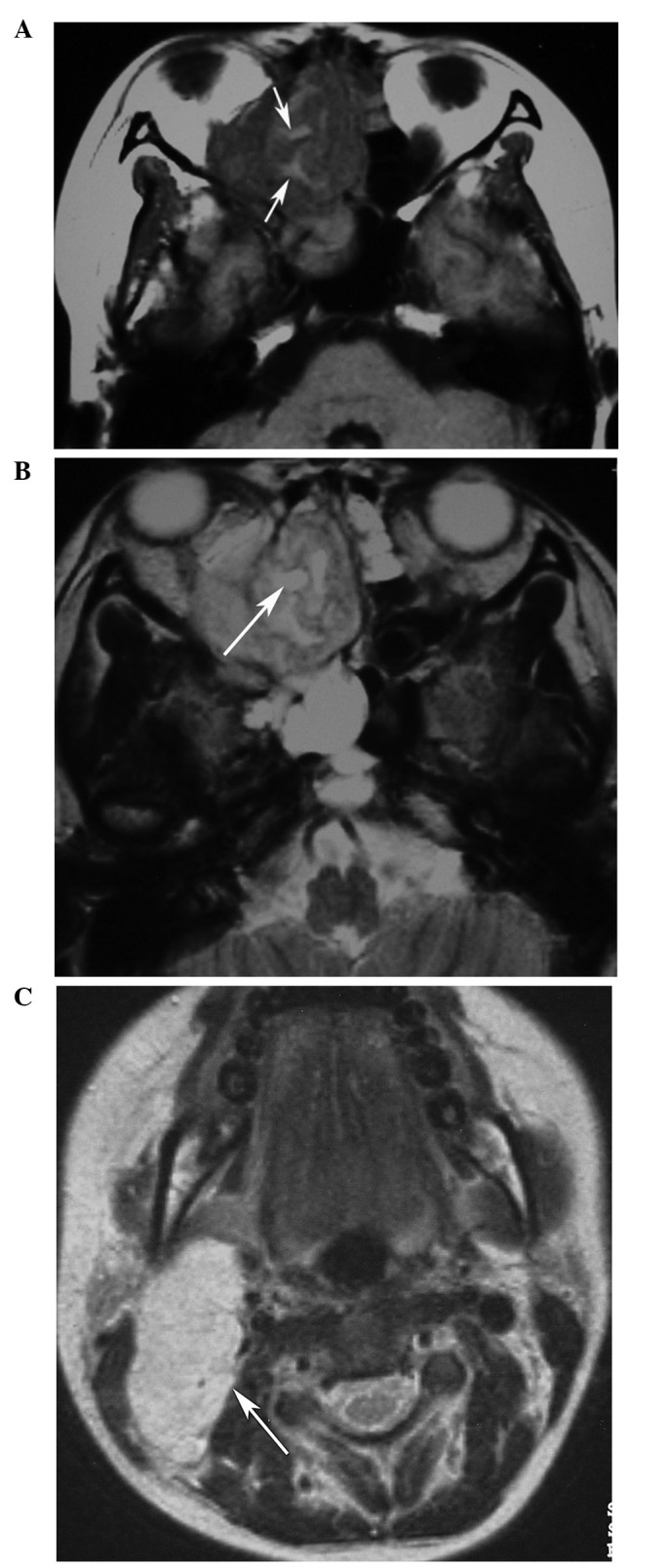
Case 3, a 17-year-old female with embryonal rhabdomyosarcoma in the ethmoid sinus. (A) Axial T1-weighted imaging (WI) shows an isointense mass originating from the right ethmoid sinus with multiple hyperintense signals (arrow). (B) Axial T2WI shows the mass with heterogeneous hyperintensity (arrow) and (C) axial T2WI demonstrates enlarged lymph nodes (arrow) involving the right side of the neck.

**Figure 3:**
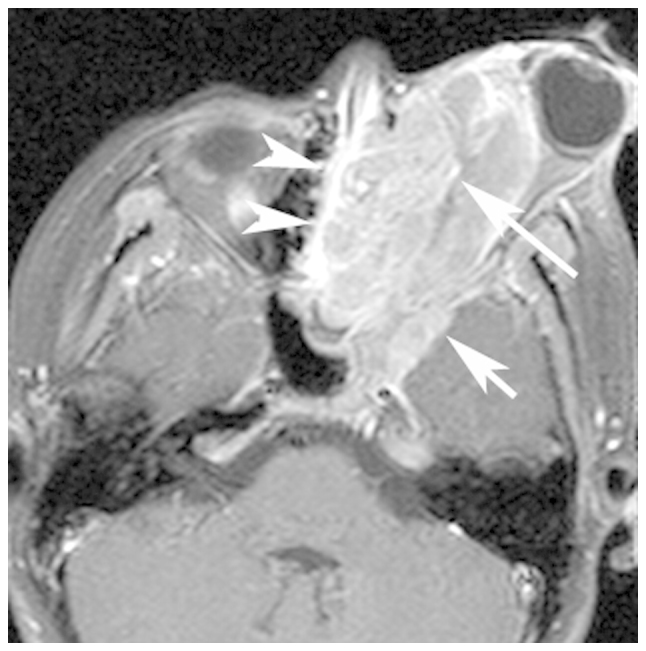
Case 8, a 13-year-old female with embryonal rhabdomyosarcoma in the orbit. Axial contrast-enhanced T1-weighted imaging shows a left orbital mass with homogeneous enhancement (long arrow) extending to the left ethmoid sinus (arrowhead) and left middle cranial fossa (short arrow). The ill-defined tumor had displaced the globe laterally and superiorly.

The 10 HNRMSs were classified into embryonal (n=8) and alveolar (n=2) subtypes, confirmed by surgery (n=2: Cases 6 and 10) and biopsy (n=8). Immunohistochemical analysis of the masses revealed characteristic positivity for desmin (n=10) and MyoD1 (n=10). The original locations of the HNRMSs were the ethmoid sinus (n=4; Figs. 1 and 2), the maxillary sinus (n=1), orbit (n=3; Fig. 3), nasopharynx (n=1) and the frontotemporal subcutaneous area (n=1). The average tumor diameter was 4.5 cm (range, 2.9–7.1 cm). On the CT (n=8) and MRI (n=9) images, 90% (9/10) of the patients exhibited ill-defined soft-tissue masses. The tumors appeared as isodense (n=6) or slightly hypodense (n=2) on the precontrast CT images, and isointense on the T1-weighted images (WI; n=9, one tumor exhibited multiple hyperintensity signals, the others exhibited a homogeneous signal). On T2WI, the tumors showed homogeneous moderate hyperintensity (n=5), homogeneous marked hyperintensity (n=1) and heterogeneous moderate hyperintensity (n=3). In addition, the masses exhibited homogeneous enhancement [n=7, one patient on post-contrast CT images, three patients on contrast-enhanced (CE) T1WI and three patients on CT and CE-T1WI] or heterogeneous enhancement (n=3, CE-T1WI only). Three embryonal RMSs (cases 1–3) originating from the ethmoid sinus demonstrated heterogeneous hyperintensity on T2WI and heterogeneous enhancement with multiple small rings, resembling nodules (Figs. 1 and 2). Unilateral or bilateral sinusitis was also observed in five patients.

Bony destruction (n=10) was ubiquitous and sclerosis was identified in two tumors originating from the ethmoid sinus (Fig. 1A). The tumors destroyed adjacent bony structures and extended into the surrounding spaces, including the paranasal sinus (n=6), nasal cavity (n=5), cranial cavity (n=5), orbit (n=4) and infratemporal fossa (n=1). Multi-cavity growth (cavities, n≥2) was identified in 70% (7/10) of patients (Fig. 3). Dural enhancement (thickness, >5 mm) (1), which was interpreted as intracranial extension, was noted in five patients (Fig. 3). Calcification and hemorrhaging were not identified in any of the patients. Unilaterally enlarged cervical lymph nodes (>1 cm in short diameter) without necrosis were observed in two patients (cases 3 and 9) and identified as metastatic by ultrasound-guided fine-needle cytology (Fig. 2C), however, no distant metastasis was identified. The CT and MRI observations of the 10 HNRMS patients together with their clinical data are summarized in Tables I and II.

## Discussion

The incidence rate of HNRMS is uncertain between males and females (5–7,14,15); however, the present study exhibited a female predominance (70%). A previous study reported that 43% of RMSs occur prior to reaching five years of age and 78% occur prior to reaching 12 years of age (6), which is consistent with the current study where the median age of patients was 16 years, with 70% of patients <20 years old. RMS exhibits a predilection for the head and neck regions, however, HNRMS in the PM, NPM and ORB locations are involved in ~50, 25 and 25% of cases, respectively (9). In the current study, the PM (60%; cases 1–5 and 9) and ORB (30%; cases 6–8) locations were the most common sites and the clinical symptoms were not specific, however, they were associated with the tumor site.

Due to the rarity of HNRMS, the majority of the available CT and MRI information is derived from small case series. Lee *et al* (7) reported that 10 HNRMSs appeared as isodense (100%; 10/10) on pre-contrast CT and homogeneously enhanced (60%; 6/10) on post-contrast CT. In addition, Hagiwara *et al* (6) presented eight HNRMSs with isointensity (37.5%) and slight hyperintensity (62.5%) on T1WI, and homogeneous (12.5%) and heterogeneous hyperintensity (87.5%) on T2WI, and heterogeneous enhancement (100%) on CE-T1WI. In the current study, the tumors appeared as isodense (75%; 6/8) or slightly hypodense (25% 2/8) on pre-contrast CT and homogeneous enhancement (100%, 4/4) was demonstrated on post-contrast CT. On MRI, the tumors demonstrated isointensity (100%; 9/9) on T1WI, homogeneously moderate to marked hyperintensity (66.7%; 6/9) or heterogeneously moderate hyperintensity (33.3%; 3/9) on T2WI, and homogeneous enhancement (66.7%; 6/9) or heterogeneous enhancement (33.3%; 3/9) on CE-T1WI. The imaging results of the HNRMS in the present study differ from previous studies. This discrepancy may be a result of the lack of HNRMS cases, however, it may be due to the different pathological subtypes.

The current histological classification for RMS includes the embryonal, alveolar and pleomorphic subtypes; the botryoid type is classified as embryonal (5). Allen *et al* (4) reported that RMSs in adults (n=26) demonstrate prominent heterogeneity and extreme hyperintensity on T2WI in the alveolar and pleomorphic subtypes. However, according to Franco *et al* (5), RMSs do not exhibit these features in children. The results of the present study revealed one embryonal RMS (11.1%; 1/9) with marked hyperintensity and three embryonal RMSs (33.3%; 3/9) with heterogeneously moderate hyperintensity on T2WI. These results indicate that HNRMS exhibit different signaling features on T2WI. Hagiwara *et al* (6) reported that the ‘botryoid sign’ on CE-MRI correlates with RMS. In the current study, nodule-shaped enhancement patterns were observed in three HNRMSs with heterogeneous hyperintensity on T2WI. All three RMSs with nodule-shaped enhancement patterns originated from the ethmoid sinus and were of the embryonal subtype. However, the remaining RMSs without nodule-shaped enhancement patterns, arising in the ethmoid sinus, maxillary sinus, orbit, nasopharynx and subcutaneous area, belonged to the embryonal (n=5) and alveolar (n=2) subtypes.

The embryonal subtype predominantly occurs in the head and neck in patients aged <10 years and accounts for 30–80% of RMSs, which are commonly composed of spindle or botryoid cells (4,7,16,17). Botryoid RMS accounts for ~5% of cases and is identified macroscopically by the presence of nodule-shaped polypoid masses, which are found in the mucosa-lined organs of the nasopharynx, paranasal sinus, genitourinary and gastrointestinal tracts (18). In the present study, embryonal RMSs with heterogeneous hyperintensity on T2WI and nodule-shaped enhancement patterns on CE-T1WI were only located in the ethmoid sinus. In addition, the signals of these three tumors were homogeneously or heterogeneously isointense with isodensity on CT, which could not be interpreted as hemorrhaging or necrosis. This indicated that the tumor contained mucus and that the tumor cells may have grown along the ethmoidal cells, which may have resulted in the existence of this mucus in the RMS, particularly in the botryoid RMS. We speculate that a mass in the ethmoid sinus, that exhibits heterogeneous hyperintensity on T2WI and nodule-shaped enhancement patterns on CE-T1WI, presents the botryoid subtype of embryonal RMS. The three embryonal RMSs with nodule-shaped enhancement patterns identified in the present study may be mixed subtypes composed of botryoid and spindle cells. However, it was not possible to identify the pathological features, as all three patients were diagnosed by biopsy, which may not have included the portion of nodule-shaped enhancement patterns.

Calcification and hemorrhaging are rare in HNRMS (2,4–7,15,19) and accordingly, these features were not present radiologically or pathologically in the current study. RMS is an aggressive malignancy, which spreads via three routes; direct extension, lymphatic metastasis and hematogenous metastasis. In total, ≤14% of patients with RMS exhibit metastatic disease at presentation (20). In addition, bony destruction has been a common imaging feature of RMS in previous studies (4–7,19,21). In the current study, bony erosion was frequently observed and sclerosis was identified in two tumors, which is a rare sign in HNRMS (5,7,19). On CT and MRI, the present study clearly demonstrated multi-cavity growth (70%) of HNRMS and three types of direct intracranial extension, including nasocranial, ORB-cranial and nasopharyngeal-cranial communication. The frequency of lymphatic metastasis was 10–20% for HNRMS and is more common in other sites (5,7,15,19,22). In addition, cervical lymph node metastases were detected in two patients (20%, 2/10) with the embryonal subtype, however, no distant metastasis was observed.

With the exception of HNRMS, other rapidly growing malignant tumors of the head and neck may be encountered in children and adults. Lymphoma usually exhibit intermediate signal intensity and homogeneous CE (11,23). In addition, NPC appear as homogeneous masses with infiltration of the adjacent soft tissue and erosion of the skull base (12). However, <20% of NPC cases occur in children (8). Furthermore, bilaterally enlarged neck nodes are common in lymphoma and NPC, and rarely occur in HNRMS (1). Osteosarcoma commonly exhibits areas of calcification, ossification or sclerosis (24). ONB is an aggressive type of neuroendocrine tumor located high in the nasal cavity. Peripheral areas of cystic degeneration and calcific foci are radiological features that are associated with ONB (13). Although these tumors exhibit such features, the CT and MRI observations are similar to the imaging manifestations of HNRMS. Thus, the differential diagnosis of malignant tumors is difficult when based solely on CT and MRI observations (25,26), therefore, the majority of masses require a biopsy to confirm the diagnosis of HNRMS.

The present study had certain limitations. Firstly, a small number of patients was included owing to the rarity of HNRMS and secondly, 80% of patients were diagnosed by biopsy. Therefore, further multicenter cooperation on the radiological diagnosis of HNRMS is required.

In conclusion, HNRMS is a rare and aggressive malignancy. MRI accurately demonstrates the location and extent of HNRMS; however, HNRMSs may exhibit certain imaging features that are similar to other tumors in the head and neck regions. The present study indicated that a tumor with heterogeneous hyperintensity on T2WI and nodule-shaped enhancement patterns in the ethmoid sinus may be considered as specific MRI features, which clearly indicate the botryoid subtype of embryonal RMS.

## Figures and Tables

**Table I:** Clinical data and imaging results of 10 patients with head and neck rhabdomyosarcoma.

Case	Age, years/gender	Pathology subtype	Tumor origin	Tumor border	Size, cm	CT (n=8)		MRI (n=9)			Lymph	Tumor extent
	
						Density	CE	T1WI	T2WI	CE-T1WI		
1	14/F	Embryonal	ES (R)	Ill-defined	7.1	Hypo	NA	Isointense	Hyper^a^	Nodular	−	MS, NC, CC
2	15/F	Embryonal	ES (R)	Ill-defined	6.9	Hypo	NA	Isointense	Hyper^a^	Nodular	−	MS, NC, O, CC
3	17/F	Embryonal	ES (R)	Ill-defined	4.0	Isodense	NA	Isointense	Hyper^a^	Nodular	+	MS, SS, NC, O
4	45/F	Embryonal	ES (L)	Ill-defined	5.6	Isodense	NA	Isointense	Hyper^b^	Homo	−	MS, NC, O, CC
5	36/M	Alveolar	MS (R)	Ill-defined	3.5	Isodense	Homo	NA	NA	NA	−	ES, NC, O, ITF
6	19/F	Embryonal	O (L)	Ill-defined	3.1	Isodense	Homo	Isointense	Hyper^b^	Homo	−	-
7	6/F	Embryonal	O (L)	Ill-defined	2.9	NA	NA	Isointense	Hyper^b^	Homo	−	-
8	13/F	Embryonal	O (L)	Ill-defined	5.7	Isodense	Homo	Isointense	Hyper^b^	Homo	−	ES, MS, CC
9	5/M	Embryonal	NP (R)	Ill-defined	3.6	NA	NA	Isointense	Hyper^c^	Homo	+	CC
10	77/M	Alveolar	S (R)	Well-defined	3.5	Isodense	Homo	Isointense	Hyper^b^	Homo	−	-

a Heterogeneously moderate,

b homogeneously moderate and

c homogeneously marked.

CT, computed tomography; MRI, magnetic resonance imaging; CE, contrast-enhancement; T1WI, T1-weighted images; T2WI, T2-weighted images; CE-T1WI, contrast-enhanced T1WI; Lymph, lymphadenopathy; F, female; M, male; ES, ethmoid sinus; MS, maxillary sinus; O, orbit; NP, nasopharynx; S, subcutaneous area; R, right; L, left; Hypo, slight hypodensity; NA, not available; Homo, homogeneous; Hyper, hyperintensity; NC, nasal cavity; CC, cranial cavity; SS, sphenoid sinus; ITF, infratemporal fossa; +, positive; −, negative.

**Table II:** Summary of the clinical and imaging results of 10 patients with head and neck rhabdomyosarcoma.

Characteristic	n (%)
Age, years	
<20	7/10 (70.0)
≥20	3/10 (30.0)
Gender	
Male	3/10 (30.0)
Female	7/10 (70.0)
Tumor origin	
Paranasal sinus	5/10 (50.0)
Orbit	3/10 (30.0)
Other	2/10 (20.0)
Pathological subtype	
Embryonal	8/10 (80.0)
Alveolar	2/10 (20.0)
Tumor border	
Ill-defined	9/10 (90.0)
Well-defined	1/10 (10.0)
Computed tomography attenuation	
Slightly hypodense	2/8 (25.0)
Isodense	6/8 (75.0)
Homogeneous	8/8 (100.0)
Homogeneous enhancement	4/4 (100.0)
T1WI	
Isointense	9/9 (100.0)
Homogeneous enhancement	6/9 (66.7)
Nodule-shaped enhancement pattern	3/9 (33.3)
T2WI	
Moderately hyperintense	8/9 (88.9)
Markedly hyperintense	1/9 (11.1)
Homogeneous	6/9 (66.7)
Heterogeneous	3/9 (33.3)
Tumor extent	
1 cavity	3/10 (30.0)
≥2 cavities	7/10 (70.0)
Bony destruction	10/10 (100.0)
Cervical lymph node metastases	2/10 (20.0)

WI, weighted images.
